# 抗表皮生长因子受体和抗血管内皮生长因子药物在非小细胞肺癌治疗中的作用

**DOI:** 10.3779/j.issn.1009-3419.2010.09.17

**Published:** 2010-09-20

**Authors:** Corey LANGER, Jean-Charles SORIA, 娟 南

**Affiliations:** 1 University of Pennsylvania, Philadelphia; 2 Institut Gustave Roussy, France; 3 天津医科大学总医院，天津市肺癌研究所，天津市肺癌转移与肿瘤微环境重点实验室

**Keywords:** 血管生成, 贝伐珠单抗, 西妥昔单抗, 联合治疗, 厄洛替尼, 酪氨酸激酶抑制剂

## Abstract

采用以铂类药物为基础联合细胞毒性药物作为非小细胞肺癌（non-small-cell lung cancer, NSCLC）的标准化一线治疗的疗效已达到相应的平台。作用于特定通路的新型分子靶向药物逐渐成为治疗NSCLC的有效药物；某些药物的Ⅲ期试验已取得阳性结果。值得注意的是，有研究证实，采用抗血管内皮生长因子（vascular endothelial growth factor, VEGF）抗体贝伐珠单抗抑制VEGF通路，以及采用表皮生长因子受体（epidermal growth factor receptor, EGFR）的小分子酪氨酸激酶抑制剂厄洛替尼或单克隆抗体（西妥昔单抗）靶向作用于EGFR通路作为一线或二线治疗方案，均可延长晚期肺癌患者的生存期。可导致肿瘤细胞存活和增殖的信号处理过程不同，为靶向作用于多种信号通路为有效的抗癌治疗策略提供了依据。因此，分子靶向药物的合理联用可能达到更好的临床疗效，并可成为对标准化疗耐药或不能耐受标准化疗的患者的可替代的治疗选择。目前的挑战在于明确应探寻哪些分子实体，以及它们的最佳联合方案。本综述旨在探讨贝伐珠单抗联合厄洛替尼抑制血管生成和EGFR信号作为治疗NSCLC的有效的非化疗方案的潜在临床疗效。其它可阻滞EGFR和血管生成通路以及互补信号通路的新型药物的联合具有独特的作用机制和较轻的毒副作用，有可能为晚期NSCLC患者的个体化治疗选择提供更多的治疗方案。

## 前言

非小细胞肺癌（non-small-cell lung cancer, NSCLC）的标准化一线治疗通常采用以铂类化疗药物为基础联合细胞毒性药物。但是，作用于调节细胞稳态和生长的特定通路的新型分子靶向药物已逐渐成为治疗NSCLC的有效药物。一些分子靶向药物正处于临床研发中，某些药物的Ⅲ期试验已取得阳性结果。贝伐珠单抗为靶向作用于血管内皮生长因子（vascular endothelial growth factor, VEGF）的单克隆抗体（monoclonal antibody, MoAb），美国批准贝伐珠单抗与紫杉醇和卡铂联合、欧洲批准贝伐珠单抗与以铂类为基础的治疗联合为晚期NSCLC患者的一线治疗选择。该批准主要基于东部肿瘤协作组（Eastern Cooperative Oncology Group, ECOG）4599试验的结果：贝伐珠单抗与化疗联合可使患者的生存期延长2个月^[1]^。人表皮生长因子受体（epidermal growth factor receptor, EGFR）的小分子酪氨酸激酶抑制剂（tyrosine kinase inhibitor, TKI）厄洛替尼和吉非替尼均被批准为晚期NSCLC患者的二线或三线治疗选择。关键的厄洛替尼试验BR.21在非指定的人群中探讨了厄洛替尼治疗复发NSCLC的疗效；与服用安慰剂组的中位总生存期（overall survival, OS）4.7个月相比，服用厄洛替尼组的中位OS为6.7个月^[2]^。采用厄洛替尼治疗亦可明显改善无进展生存期（progression-free survival, PFS）、肿瘤有效率（response rate, RR）、疾病控制率、肿瘤相关症状以及患者生活质量^[3]^，故其被批准用于再发肺癌患者。大规模的ISEL（Iressa Survival Evaluation in Lung Cancer）试验结果显示，在全部人群中，与安慰剂相比，吉非替尼单一疗法并未带来具有统计学差异的生存获益，其后，除了用于部分临床试验外，吉非替尼在美国和欧洲不再使用，但特定的亚组患者可获益于吉非替尼治疗^[4]^。最近有研究报道，在晚期NSCLC中，与单独化疗相比，西妥昔单抗联合标准化疗具有生存优势^[5]^；尽管该药物尚未被批准用于NSCLC，但这些结果与之前有关比较EGFR TKIs联合化疗与单独化疗的研究结果大相径庭。

靶向药物的独特作用机制及其喜人的毒副作用谱为对标准化疗耐药或无法耐受标准化疗的患者提供了可替代的治疗选择。目前的挑战在于明确应探寻哪些分子实体，以及靶向作用于它们的最佳方案。

## 新型的非细胞毒性方案治疗非小细胞肺癌的依据

在晚期肺癌患者中，与标准化疗相比，分子靶向药物的联合使用可能产生额外的肿瘤反应、生存获益和增高的耐受性（表 1）^[6-25]^。以靶向作用于生物过程和信号通路作为各种恶性肿瘤治疗选择的假定临床疗效，导致直接作用于肿瘤细胞或肿瘤脉管系统内特定分子实体的分子靶向药物的研发。这些方案的大部分临床进展为靶向作用于EGFR和VEGF信号通路（图 1）。VEGF可与3种不同的受体酪氨酸激酶结合；其中，血管内皮细胞中VEGF受体（VEGFR）-2信号的过度激活是肿瘤血管生成的主要介导因子^[26, 27]^。EGFR信号与对肿瘤形成和转移至关重要的许多过程相关，包括细胞存活、增殖、粘附、分化、迁移、转化和运动^[28, 29]^。VEGF和EGF均可激活调节基因表达和细胞增殖的Ras/ Raf/MEK信号通路^[27, 29]^。VEGF和EGF亦可促进细胞存活，主要靶标分别为内皮和肿瘤细胞。因此，VEGF和EGFR信号在肿瘤形成中发挥互相独立但互为补充的作用。

**1:** 作为晚期非小细胞肺癌治疗选择的分子靶向药物指定联合的临床研发 Clinical development of selected combinations of molecular targeted agents as treatment options for advanced non-small-cell lung cancer

药物联合	临床前	Ⅰ/Ⅱ期	Ⅲ期
EGFR+VEGF	√^[6, 7]^	√^[15, 16]^	在研^[23, 24]^
EGFR+mTOR	√^[8-10]^	在研^[17-19]^	—
EGFR+IGF-1R	√^[11]^	在研^[20]^	在研^[25]^
VEGF+VDA	√^[12, 13]^	在研^[21]^	—
mTOR+IGF-1R	√^[14]^	在研^[22]^	
Abbreviations: EGFR=epidermal growth factor receptor; IGF-1R=insulin-like growth factor receptor-1; mTOR=mammalian target of rapamycin; NSCLC= non-small-cell lung cancer; VDA=vascular disrupting agents; VEGF=vascular endothelial growth factor. Note: Reprinted with permission from the copyright holder©CIG Media Group, LP 缩写：EGFR：表皮生长因子受体；IGF-1R：胰岛素样生长因子Ⅰ型受体；mTOR：哺乳类动物雷帕霉素靶蛋白；NSCLC：非小细胞肺癌；VDA：血管破坏剂；VEGF：血管内皮生长因子。 注：本图得到版权所有者©CIG Media Group, LP复制许可			

**1:**
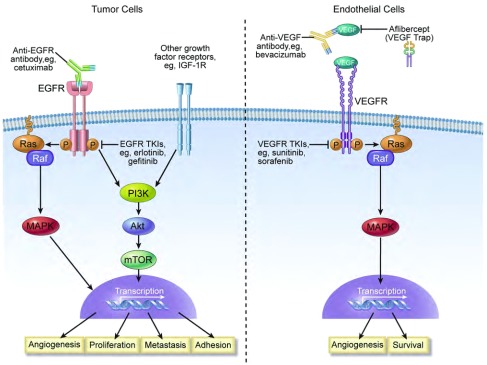
EGFR和VEGFR激活的下游信号转导和生物过程以及抑制这些信号通路组分的靶向治疗。肿瘤细胞中EGFR的激活会引起受体激酶区域内酪氨酸残基的磷酸化以及后续的Ras/Raf/MAPK或PI3K/Akt/mTOR通路的激活，这将导致血管生成、细胞增殖、生长、转移和粘附相关基因的核激活。其它生长因子受体通路（如IGF-1R）的激活亦可通过PI3K和其它下游组分而传递信号，进而导致基因转录。内皮细胞中VEGFR的激活亦可活化Ras/Raf/MAPK通路，并调节基因表达和细胞存活。有两种策略可抑制EGFR介导的信号：可与细胞内激酶区域结合的小分子TKI（如，厄洛替尼和吉非替尼）或可结合于细胞外并阻止配体结合的单克隆抗体，如西妥昔单抗。可与VEGF配体直接结合并阻止受体激活的抗VEGF抗体（如，贝伐珠单抗）或VEGFR的小分子TKI（如，舒尼替尼和索拉非尼）亦可同样抑制VEGF介导的下游信号。 Signal transduction and biologic processes downstream of egfr and vegfr activation and targeted therapies that inhibit components of these signaling pathways. Activation of EGFR on tumor cells leads to phosphorylation of tyrosine residues in the kinase domain of the receptor and subsequent activation of the Ras/Raf/MAPK or the PI3K/Akt/mTOR pathways, resulting in nuclear activation of genes related to angiogenesis, cell proliferation, growth, metastasis, and adhesion. Activation of other growth factor receptor pathways (eg, IGF-1R) also signal via PI3K and other downstream components, resulting in gene transcription. Activation of VEGFR on endothelial cells also activates the Ras/Raf/MAPK pathway and regulates gene expression and survival. Two strategies are available to inhibit EGFR-mediated signaling: small-molecule TKIs (eg, erlotinib and gefitinib) that bind to the intracellular kinase domain or monoclonal antibodies such as cetuximab, which bind extracellularly and prevent ligand binding. VEGF-mediated downstream signaling can be similarly abrogated via anti-VEGF antibodies (eg, bevacizumab), which bind directly to the VEGF ligand and prevent receptor activation, or by small-molecule TKIs (eg, sunitinib and sorafenib) of the VEGFR.

临床前研究证实抑制EGFR信号和血管生成的药物的联用可产生协同的抗肿瘤效应^[6, 7]^。在肿瘤细胞中，VEGF和EGF间存在功能关系。EGFR信号可上调许多血管生成因子的表达，包括VEGF、白介素8和碱性成纤维细胞生长因子（fibroblast growth factor, FGF)^[29, 30]^。EGFR亦可通过增加源自肿瘤细胞的促血管生成因子的合成和释放而间接调节血管生成。因此，EGFR信号对肿瘤细胞既有直接作用（如，存活和增殖），又有间接作用（通过新的血管形成供给氧气和营养成分）。此外，在肿瘤进展中，EGF是VEGF表达的关键调节因子；阻断EGFR信号可导致EGFR通路非依赖性的VEGR表达上调。有猜测认为当采用抗EGFR药物治疗肿瘤时，这种EGFR非依赖性的VEGF表达上调可能是肿瘤逃逸的机制。这些资料为联合靶向作用于EGFR信号和血管生成通路以治疗NSCLC提供了强有力的依据^[6, 7, 31, 32]^。

为了使晚期NSCLC的临床疗效最大化，靶向治疗的联合应证实以下内容：（1）许多通路（如，EGFR和/或VEGF/血管生成信号通路）抑制的最大化，（2）毒副作用和治疗相关不良事件（adverse event, AE）的最小化，（3）剂量与方案的灵活性。在NSCLC中，联合靶向作用于EGFR和VEGF理论上可以通过采用恰当的单一多靶点药物或通过联合不同的单一靶点药物来实现。

## 抗血管内皮生长因子和抗表皮生长因子受体靶向药物的联合

在NSCLC中，联合靶向作用于VEGF和EGFR信号的依据^[31, 32]^是其在克服肿瘤耐药性能力方面具有潜在协同作用，而仅阻断这两条通路中的一条有可能导致肿瘤耐药性^[29]^。两种单一靶点药物联用应考虑到各种药物的独立给药剂量方案，从而可提高对VEGF/血管生成信号通路和EGFR信号通路的最大化抑制。这与多靶点单一药物形成对比，多靶点单一药物无法以恰当的给药剂量实现对每一靶点的最大化抑制。分子靶向药物可能需要剂量下调（暂时的或永久的）以控制毒副作用。但是，多靶点药物的剂量下调可能削弱对所有靶点的抑制作用，无论这些靶点对于AE的发生是否重要。相反，两种单一靶点药物的联合可通过剂量下调或暂时停药以更好地控制一种药物的相关毒副作用，且不影响另一药物的疗效。两种单一靶点药物的叠加作用或者甚至协同作用应考虑到治疗方案的灵活性、患者的个体化治疗及可能的更佳疗效。例如，在某些病例中，相继靶向作用比同时靶向作用于两条通路的疗效更好。两种单一靶点药物的联用可通过单一药物的间歇性给药实现药效分离。有研究推断采用EGFR TKI和另一种药物的“交叉”疗法可提高疗效，并可克服同时使用EGFR TKI和化疗时出现的耐药性^[33, 34]^。

可作用于多种特异通路的单一靶点药物的联合方案亦可应用综合杀伤力的概念，即对任何两种基因的单独突变不起作用，但可使携带两种基因突变的细胞无法存活。在癌症治疗中，对多种基因的抑制是对多种致癌突变的综合杀伤，因此可杀伤仅携带相应突变的细胞（如，肿瘤细胞）和多余的正常细胞；这种方案可成为疗效指数改善的疗法^[35]^。综合杀伤力疗法为靶向治疗选择提供了一种独特的方案；目前的挑战是明确分子靶标，当联合阻滞这些分子靶标时会引起肿瘤细胞死亡。新兴的证据表明靶向作用于多条通路的疗效优于靶向作用于单一通路内的多个组分，单一靶向药物的合理联合在带来最佳临床获益的同时也使患者的耐受性更好^[31, 32, 36]^。

## 非小细胞肺癌中血管内皮生长因子和表皮生长因子受体靶向作用的临床验证

目前，对于NSCLC和其它肿瘤类型，数个血管生成抑制剂正处于临床研发阶段^[37, 38]^。在NSCLC中，临床进展最大的血管生成抑制剂为人源化抗VEGF MoAb贝伐珠单抗^[39]^，其次为aflibercept（VEGF Trap）。目前，aflibercept与多西紫杉醇的联合正处于Ⅲ期临床试验中^[40]^。在针对NSCLC一线治疗的一项随机Ⅲ期试验中，与单独化疗（紫杉醇和卡铂）相比，贝伐珠单抗联合化疗的中位OS延长2个月^[1]^。这一获益具有统计学意义和一致性，1年和2年生存率亦是如此。同样，AVAiL（Avastin in Lung Cancer）试验达到了主要终点，即：随机接受贝伐珠单抗联合化疗（顺铂/吉西他滨）的患者较单独化疗的患者的PFS显著改善；RR为次要终点，随机分至治疗组的患者较对照组患者的RR亦明显增加（对照组、贝伐珠单抗7.5 mg/kg组和贝伐珠单抗15 mg/kg组的PFS分别为6.1个月、6.7个月和6.5个月；RR分别为20%、34.1%和30.4%）^[41]^。但是，在该试验中，贝伐珠单抗的PFS获益并未转化为显著的OS获益（次要终点），可能是由于不同的二线治疗药物的大量使用^[42]^。

贝伐珠单抗联合以铂类药物为基础的化疗的临床获益验证了抗血管生成方案治疗晚期或转移性NSCLC的疗效。但是，抗血管生成治疗的挑战之一是晚期实体瘤的脉管系统可能不同，因此较早期疾病更难治疗^[43]^。肿瘤脉管系统的异质性有可能限制抗血管生成药物在晚期或转移性NSCLC中的疗效，这为其它抗癌治疗与抗血管生成药物的联合提供了依据^[44]^。对于晚期NSCLC患者，贝伐珠单抗单一疗法的临床获益适中（modest），但是数据有限。在采用贝伐珠单抗治疗晚期NSCLC的一项随机Ⅱ期试验中，疾病进展后从对照组转移至贝伐珠单抗单一疗法组的19例患者未见肿瘤治疗反应；但是，改变治疗后生存期达12个月的患者占47%，26%的患者达疾病稳定^[45]^。

EGF在肿瘤发生中的关键作用以及EGFR在原发NSCLC中的频繁过表达^[28, 29, 46]^已促使了靶向作用于这一信号通路的药物的研发^[47, 48]^。EGFR TKI吉非替尼和厄洛替尼在NSCLC中的疗效已被广泛研究；在这些研究结果中，厄洛替尼为各患者亚组带来了更多获益^[48]^。尽管厄洛替尼联合化疗作为晚期NSCLC的一线治疗并未呈现生存获益^[49, 50]^，但厄洛替尼可延长一线或二线化疗失败后的非指定患者的生存期^[2]^。吉非替尼联合化疗一线治疗晚期NSCLC或作为预处理患者的单一药物均未呈现生存获益^[4]^。但是，INTEREST（Iressa NSCLC Trial Evaluating Response and Survival Against Taxotere）试验的近期数据显示，对于采用以铂类药物为基础的化疗预处理的NSCLC患者，吉非替尼并不劣于多西他赛^[51]^。此外，IPASS（IRESSA Versus Carboplatin/Paclitaxel in Asia）试验的早期报告评估了吉非替尼或卡铂/紫杉醇一线治疗特定亚裔患者人群的疗效，结果显示，对于所有患者，在PFS和RR方面，吉非替尼优于卡铂/紫杉醇。在伴有*EGFR*突变阳性的肿瘤患者中，吉非替尼治疗组的PFS明显优于卡铂/紫杉醇治疗组；但是，对于EGFR阴性的肿瘤患者，化疗联合治疗组的PFS更佳^[52]^。这些数据已于近日发表于新英格兰医学杂志（*New England Journal of Medicine*）上。有2项Ⅲ期试验评估了EGFR MoAb西妥昔单抗一线治疗晚期NSCLC患者的疗效，尽管该药尚未批准用于治疗NSCLC。在第1项试验FLEX（First-Line in Lung Cancer with Erbitux）中，西妥昔单抗联合化疗（顺铂/长春瑞滨[CV]）较单一化疗具有生存优势（中位OS：西妥昔单抗联合CV为11.3个月*vs*单一CV为10.1个月，*P*=0.04）^[5]^。但是，第2项小型试验显示，西妥昔单抗联合卡铂/紫杉烷较单一化疗的OS无明显改善。接受西妥昔单抗联合化疗的患者的中位OS为9.7个月，接受单一化疗的患者的中位OS为8.4个月（*P*=0.17）^[53]^。

综上，这些结果提示，贝伐珠单抗联合厄洛替尼以抑制血管生成和EGFR信号通路有可能成为治疗晚期NSCLC的有效方案。

## 厄洛替尼联合贝伐珠单抗：非小细胞肺癌治疗的非化疗选择

厄洛替尼联合贝伐珠单抗可作用于恶性肿瘤发生必不可少的2条信号通路；因此，联合给予两种药物可能为晚期疾病患者带来额外的临床获益。这种联合亦可同时抑制2条不同但互补的生物通路中的受体（EGFR）和配体（VEGF），并不会加重常见的毒副作用。一项有关至少曾接受过1次化疗方案患者的非对照Ⅰ/Ⅱ期研究最先证实了厄洛替尼联合贝伐珠单抗对晚期非鳞型NSCLC的临床获益^[15]^。两药间未见药物代谢动力学交叉反应，AE多为轻至中度（包括皮疹、腹泻、感染、血尿和蛋白尿）且易于处理。采用厄洛替尼联合贝伐珠单抗的患者的中位OS为12.6个月，非指定患者（对EGFR表达情况无要求）的中位PFS为6.2个月。近日后续的3个分组的Ⅱ期研究结果被报道，该研究将患者分为化疗（多西紫杉醇或培美曲塞）+安慰剂组、贝伐珠单抗+化疗组和厄洛替尼+贝伐珠单抗组（表 2）^[16]^。厄洛替尼+贝伐珠单抗组患者的1年生存率为57.4%，贝伐珠单抗+化疗组为53.8%，单一化疗组为33.1%。厄洛替尼+贝伐珠单抗组的中位OS为13.7个月，贝伐珠单抗+化疗组为12.6个月，单一化疗组为8.6个月。与标准化疗相比，厄洛替尼+贝伐珠单抗的耐受性亦较好。厄洛替尼+贝伐珠单抗组中由于AE中断治疗的患者仅占13%，单一化疗组为24%，贝伐珠单抗+化疗组为28%。

**2:** 厄洛替尼+贝伐珠单抗的Ⅱ期研究中3个治疗组的疗效和耐受性结果^[16]^ Efficacy and tolerability outcomes of the 3 treatment arms of the phase Ⅱ study of erlotinib plus bevacizumab^[16]^

疗效/耐受性指标	厄洛替尼+贝伐珠单抗	贝伐珠单抗+化疗	单一化疗
中位OS（月）	13.7	12.6	8.6
1年生存率（%）	57.4	53.8	33.1
6个月PFS率（%）	33.6	30.5	21.5
不良反应导致治疗中止的发生率（%）	13	28	24
Abbreviations: OS=overall survival; PFS=progression-free survival. Note: Reprinted with permission from the copyright holder ©CIG Media Group, LP 缩写：OS：总生存期；PFS：无进展生存期。 注：本图得到版权所有者©CIG Media Group, LP复制许可			

在一项评估厄洛替尼+贝伐珠单抗一线治疗未曾接受过治疗的患者的Ⅱ期研究中，75%的患者在第6周未见疾病进展，且联合方案的耐受性好，3/4级AE的发生率较低^[54]^。因此，厄洛替尼联合贝伐珠单抗是具有临床疗效且耐受性好的治疗方案，允许灵活的给药剂量，从而可更好地控制治疗相关的毒副作用。而且，单一药物的灵活给药剂量可控制其相关毒副作用，且不会削弱另一药物的疗效。这一策略已成功应用于厄洛替尼+贝伐珠单抗二线治疗NSCLC的临床试验中^[15]^。这些研究的振奋人心的结果表明，厄洛替尼联合贝伐珠单抗将成为针对晚期NSCLC患者的一种新的且具有临床疗效的非化疗治疗选择。

## 厄洛替尼联合贝伐珠单抗在非小细胞肺癌治疗中的展望：在研的Ⅲ期试验

上述Ⅱ期研究结果表明，与含有多西紫杉醇或培美曲塞的方案相比，厄洛替尼联合贝伐珠单抗的毒副作用谱比较令人满意^[16]^。由于毒副作用多与老年人（≥70岁）相关，因此厄洛替尼联合贝伐珠单抗有可能为非鳞型NSCLC老年患者提供更佳的治疗选择。最近，一项旨在评估厄洛替尼联合贝伐珠单抗治疗未曾接受过治疗的晚期NSCLC老年患者（≥70岁）疗效的开放Ⅱ期试验正在验证这一猜想^[55]^。另一项有关厄洛替尼联合贝伐珠单抗的试验为BeTa Lung trial，其为一项有关厄洛替尼联合贝伐珠单抗二线治疗晚期NSCLC的随机、多中心、安慰剂对照、双盲Ⅲ期研究。本试验中，患者被随机分为厄洛替尼150 mg/d+静脉注射安慰剂每三周一次或厄洛替尼150 mg/d+贝伐珠单抗15 mg/kg每三周一次。主要终点为OS，次要终点包括PFS、安全性以及对临床疗效具有预测价值的EGFR生物标记物的鉴定^[23]^。该试验的初步结果证实了厄洛替尼联合贝伐珠单抗的临床疗效具有前景意义，厄洛替尼+贝伐珠单抗组的RR较厄洛替尼+安慰剂组明显改善（厄洛替尼+贝伐珠单抗组为12.6% *vs*厄洛替尼+安慰剂组为6.2%，*P*=0.006）。厄洛替尼+贝伐珠单抗组的中位PFS为3.4个月，而厄洛替尼+安慰剂组为1.7个月[危险比（hazard ratio, HR）=0.62, *P* < 0.000 1]。但是，两组的中位OS相当（厄洛替尼+贝伐珠单抗组为9.3个月；厄洛替尼+安慰剂组为9.2个月；HR=0.97；*P*=0.75）。该试验两组中的大部分患者接受了后续治疗，从而在某种程度上未达到主要终点OS^[56]^。在ATLAS肺癌试验中，患者接受一种基于铂类化疗方案的指定方案+贝伐珠单抗一线治疗NSCLC。4个周期后，患者随机继续服用贝伐珠单抗+安慰剂或贝伐珠单抗+厄洛替尼^[24]^。该试验的初步结果报道于2009年美国临床肿瘤学会（American Society of Clinical Oncology, ASCO）会议上。化疗+贝伐珠单抗治疗后，采用厄洛替尼+贝伐珠单抗可显著改善PFS，且安全性与单一药物已知的安全性一致。随机分组后，厄洛替尼+贝伐珠单抗组的中位PFS为4.8个月，而贝伐珠单抗+安慰剂组为3.7个月（HR=0.722; *P*=0.001 2）^[57]^。总生存期的结果尚需等待。这些研究结果有助于阐释厄洛替尼联合贝伐珠单抗在晚期NSCLC维持和二线治疗中的作用。

## 非小细胞肺癌中分子靶向药物的联合

目前有许多药物处于临床前或临床研发阶段，它们对数个激酶的受体和细胞内靶标具有多靶点靶向作用^[36, 37]^。这些多靶点药物为靶向作用于一种或多种VEGFR以及其它受体的小分子TKI。其它的靶标受体包括EGFR、血小板源性生长因子受体β（platelet-derived growth factor receptor β, PDGFR-β）、FGF受体、FLT-3和c-Kit（表 3）。舒尼替尼可抑制VEGFR-2（血管生成的主要调节因子）、PDGFR-β、FLT-3和c-Kit，而西地尼布（AZD2171）可抑制VEGFR-1–3、PDGFR-β和c-Kit^[36, 47]^。索拉非尼具有更广泛的机制，包括抑制多种受体的酪氨酸激酶（VEGFR-1–3、PDGFR-β、FLT-3和c-Kit）以及丝氨酸/苏氨酸Raf激酶^[58]^。凡德他尼（ZD6474）是作用于VEGFR-2和EGFR的小分子TKI，亦可抑制RET酪氨酸激酶^[36]^。有研究显示，新型pan-HER/VEGFR抑制剂BMS-690514对NSCLC细胞系具有抗增殖和促凋亡作用^[59]^；有临床研究显示，携带T790M突变的肿瘤患者采用BMS-690514治疗后呈现部分缓解^[60]^。针对NSCLC治疗的处于在研早期的其它靶向药物包括pan-HER TKI[PF-00299804和来那替尼（HKI-272）]和XL647。pan-HER TKI为ErbB受体的不可逆性抑制剂，XL647可抑制多种酪氨酸激酶（EGFR、HER2、VEGFR-2和EphB4）^[61]^。

**3:** 与HER或VEFR通路相互作用的指定的分子靶向药物 Selected molecular targeted agents interacting with the her or vegf pathways

靶向药物	VEGF-A配基	VEGFR	EGFR	其它靶标
贝伐珠单抗^[1]^	√			
厄洛替尼^[2]^			√	
吉非替尼^[52]^			√	
索拉非尼^[58]^		√		PDGFR-*β*, FLT-3, c-Kit, Raf kinase
舒尼替尼^[36, 37]^		√		PDGFR-*β*, FLT-3, c-Kit
西地尼布/AZD2171^[36, 37]^		√		PDGFR-*β*, c-Kit
凡德他尼^[36, 37]^		√	√	RET
BMS-690514^[59]^		√	√	Pan-HER
PF-00299804^[61]^			√	Pan-HER
来那替尼/HKI-272^[61]^			√	HER2
XL647^[61]^		√	√	HER2, EphB4
Abbreviations: EGFR=epidermal growth factor receptor; PDGFR=platelet-derived growth factor receptor; VEGF=vascular endothelial growth factor; VEGFR=vascular endothelialb growth factor receptor. Note: Reprinted with permission from the copyright holder ©CIG Media Group, LP 缩写：EGFR：表皮生长因子受体；PDGFR：血小板源性生长因子受体；VEGF：血管内皮生长因子；VEGFR：血管内皮生长因子受体。 注：本图得到版权所有者©CIG Media Group, LP复制许可				

多靶点药物之所以引人注目是因为它们可以同时作用于多个靶标，且便于口服。Ⅱ期试验为多靶点药物单药治疗晚期NSCLC提供了令人鼓舞的数据^[62-64]^。但是，某些药物的Ⅲ期疗效结果令人失望，且尚无其它药物的结果。例如，E2501是一项采用独特的随机终止设计在疾病稳定的患者中比较索拉非尼与安慰剂治疗晚期NSCLC疗效的随机Ⅱ期试验，其结果显示索拉非尼可延长高强度预处理的疾病进展较慢的患者的PFS（索拉非尼组的中位PFS为3.6个月*vs*安慰剂组为1.9个月，*P*=0.001）。但是，仅约25%的患者可随机分组，大多数患者在使用索拉非尼的2个月内出现疾病进展^[65]^。此外，一项有关索拉非尼联合紫杉醇和卡铂一线治疗NSCLC的Ⅲ期试验（Evaluation of Sorafenib, Carboplatin and Paclitaxel Efficacy, ESCAPE）被过早终止^[66]^，其原因为一项有计划的中期分析显示索拉非尼+化疗与单一化疗相比无生存获益（表 4）^[67]^。随后，由于安全性问题远远超过疗效，一项有关西地尼布联合化疗一线治疗晚期NSCLC的Ⅱ/Ⅲ期试验（BR.24）也被中止^[68]^。毒副作用呈剂量依赖性，且30 mg组的毒副作用较45 mg组弱。因此，加拿大国家癌症学会肺癌组展开了一项比较西地尼布20 mg与安慰剂联合卡铂/紫杉醇疗效（研究名为BR.29）的新的随机试验^[69]^。

**4:** 评估化疗+索拉非尼与单一化疗治疗未曾接受过治疗的晚期非小细胞肺癌患者的疗效的Ⅲ期试验（ESCAPE试验）结果^[64]^ Results from the phase Ⅲ trial (escape trial) evaluating chemotherapy plus sorafenib versus chemotherapy alone in patients with previously untreated advanced non-small-cell lung cancer^[64]^

疗效指标	卡铂/紫杉醇+索拉非尼	卡铂/紫杉醇+安慰剂	危险比
OS（月）	10.7	10.6	1.16; 95%CI: 0.95-1.43; *P*=0.93
PFS（月）	5.1	5.4	1.0; 95%CI: 0.85-1.18; *P*=0.51
ORR（CR+PR）(%)	30	2.4	—
各组织学类型的OS（月）			
鳞癌	8.9	13.6	1.81; 95%CI: 1.19-2.74
非鳞癌	11.5	10.3	0.98; 95%CI: 0.78-1.24
Abbreviations: CR=complete response; HR=hazard ratio; NSCLC=non–small-cell lung cancer; ORR=objective response rate; OS=overall survival; PFS=progression-free survival; PR=partial response. Note: Reprinted with permission from the copyright holder ©CIG Media Group, LP 缩写：CR：完全缓解；HR：危险比；NSCLC：非小细胞肺癌；ORR：客观缓解率；OS：总生存期；PFS：无进展生存期；PR：部分缓解。 注：本图得到版权所有者©CIG Media Group, LP复制许可			

索拉非尼和西地尼布的随机试验结果令人失望，使人们针对晚期NSCLC的治疗提出了以下问题：

● pan-VEGFR抑制剂与化疗联合更好，还是与其它分子靶向药物联合更有效？

● 这些药物失败的原因是否是由于对血管生成通路的过度靶向作用，这样可能会限制疗效并增加毒副作用，尤见于鳞癌？

● 靶向作用于多种通路是否优于靶向作用于特定通路内的多个组分？是否优于多种具有更多有限的靶标特异性的药物的联合？

凡德他尼，可以抑制VEGFR、EGFR和RET，有随机Ⅱ期试验评估了其对晚期NSCLC患者的疗效。这些试验数据显示，当凡德他尼单一疗法或与化疗联用时，凡德他尼的治疗与PFS（而不是OS）的延长相关（表 5）^[62, 64]^。这些发现引发了在晚期NSCLC患者中评估凡德他尼单一疗法或与化疗联合的疗效的Ⅲ期试验。最近完成的试验比较了凡德他尼+多西紫杉醇与多西紫杉醇对一线治疗失败的患者的疗效，凡德他尼与厄洛替尼作为二/三线治疗的疗效，凡德他尼+培美曲赛与安慰剂+培美曲赛对一线治疗失败的患者的疗效，以及凡德他尼与安慰剂对化疗和EGFR TKI治疗失败的患者的疗效^[70]^。这些试验中的3项试验的初步结果报道于2009年ASCO年会上（表 6）^[71-74]^。ZODIAC（Zactima in Combination With Docetaxel in NSCLC；凡德他尼+多西紫杉醇*vs*多西紫杉醇单一疗法）试验达到了其主要终点，即：PFS的改善具有统计学意义。但是，ZEAL（Zactima Efficacy With Alimta in Lung Cancer；凡德他尼+培美曲赛*vs*培美曲赛单一疗法）和ZEST（Zactima Efficacy When Studied Versus Tarceva；凡德他尼*vs*厄洛替尼）试验均未达到PFS延长的主要终点。3项试验中，含凡德他尼组的OS获益均无统计学意义。因此，凡德他尼对再发NSCLC患者的疗效有待进一步阐明。

**5:** 凡德他尼二线治疗晚期非小细胞肺癌的已完成的Ⅱ期试验结果 Results of completed phase Ⅱ trials of vandetanib as second-line therapy for advanced non–small-cell lung cancer

	中位PFS (周）	中位OS (月）	ORR (%)
试验3 V (300 mg) Versus G (250 mg)^[60]^; *n*=168	V:11	V: 6.1	V:8 G:1
	G:8.1	G:7.4	
	HR=0.69;*P*=0.025^a^	HR=1.19;*P*=0.34	
试验6 V (100 mg)+D (75 mg/m^2^) Versus V (300 mg)+D (75 mg/m^2^) Versus Placebo+D (75 mg/m^2^)^[62]^; *n*=127	V (100)+D: 18.7; ^b^HR (V100)=0.64; *P*=0.074^a^ V (300)+D: 17.0; ^b^HR (V300)=0.83; *P*=0.416 D: 12.0	V(100)+D:13.1	V(100)+D:26
		V (300)+D: 7.9	V(300)+D:18
		D:13.4	D:12
		*P*=NS	
^a^Study met its primary endpoint significance criteria. ^b^HR is compared with placebo + docetaxel. Abbreviations: D=docetaxel; G=gefitinib; HR=hazard ratio; NS=not significant; NSCLC=non–small-cell lung cancer; OS=overall survival; ORR=overall response rate; PFS=progression-free survival; V=vandetanib. Note: Reprinted with permission from the copyright holder ©CIG Media Group, LP ^a^研究达到主要终点的意义标准。^b^HR与安慰剂+多西紫杉醇组比较。缩写：D：多西紫杉醇；G：吉非替尼；HR：危险比；NS：无意义；NSCLC：非小细胞肺癌；OS：总生存期；ORR：总有效率；PFS：无进展生存期；V：凡德他尼。 注：本图得到版权所有者©CIG Media Group, LP复制许可			

**6:** 评估凡德他尼单一疗法或与化疗联合治疗晚期非小细胞肺癌患者的在研Ⅲ期试验 Ongoing phase Ⅲ trials evaluating vandetanib as a single agent or in combination with chemotherapy in patients with advanced non– small-cell lung cancer

试验名称（几线治疗）	纳入的患者/患者人数	治疗组	结果
ZEPHYR (三/四线）	曾采用EGFR抑制剂治疗的晚期NSCLC患者；*n*=930	V (300 mg/d) *vs*. placebo	结果未知（主要重点：OS; 次要重点：PFS, DR)^[78]^
ZEST (二线)	一线治疗失败的晚期NSCLC患者; *n*=1 150	V (300 mg/d) *vs*. E (150 mg/d)	V *vs*. E的PFS或OS的改善无统计学意义（PFS:HR=0.98;*P*=0.721;OS: HR=1.01;*P*=0.830))^[79]^
ZEAL (二线)	一线治疗失败的晚期NSCLC患者；*n*=510	V (100 mg/d)+P (500 mg/m^2^, 每三周一次）*vs*. placebo+P	V+P的PFS或OS的改善无统计学意义（PFS: HR= 0.86; *P*=0.108; OS: HR=0.86; *P*=0.219) ^[80]^
ZODIAC (二线)	一线治疗失败的晚期NSCLC患者；*n*=1 380	V (100 mg/d)+D (75 mg/m^2^, 每三周一次)*vs*. placebo+D	V+D *vs*. D单一疗法PFS明显延长（HR=0.79; *P* < O.001);V+D的OS无明显延长（HR=0.91;*P*=0.196)^[81]^
Abbreviations: D=docetaxel; DR=duration of response; E=erlotinib; EGFR=epidermal growth factor receptor; NSCLC=non-small-cell lung cancer; OS=overall survival; P=pemetrexed; PFS=progression-free survival; RR=response rate; V=vandetanib; ZEAL=Zactima Efficacy With Alimta in Lung Cancer; ZEPHYR=Zactima Efficacy Trial for NSCLC Patients With History of EGFR-TKI and Chemoresistance; ZEST=Zactima Efficacy Study Versus Tarceva; ZODIAC=Zactima in Combination With Docetaxel in NSCLC. Note: Reprinted with permission from the copyright holder ©CIG Media Group, LP 缩写：D：多西紫杉醇；DR：反应持续时间；E：厄洛替尼；EGFR：表皮生长因子受体；NSCLC：非小细胞肺癌；OS：总生存期；P：培美曲塞；PFS：无进展生存时间；RR：有效率；V：凡德他尼；ZEAL：凡德他尼+培美曲赛*vs*培美曲赛单一疗法在肺癌中的疗效研究；ZEPHYR：凡德他尼在曾采用EGFR-TKI治疗和对化疗耐受的患者中的疗效试验；ZEST：凡德他尼*vs*厄洛替尼的疗效研究；ZODIAC：凡德他尼+多西紫杉醇*vs*多西紫杉醇单一疗法在NSCLC中的疗效研究。 注：本图得到版权所有者©CIG Media Group, LP复制许可			

目前，针对NSCLC治疗，尚无大样本、随机、头对头的研究证实多靶点药物较单一靶点药物、单一疗法或与其它单一靶点药物联合的临床疗效更好，多靶点药物的在研试验的结果有助于明确这一观点及指导未来的治疗策略。

## 表皮生长因子受体的双重靶向作用

有研究者建议采用抗EGFR抗体和EGFR TKI以实现对NSCLC中EGFR信号通路的双重靶向作用^[75]^。通过这两种药物的联合，可能同时纵向抑制EGFR并增强阻滞下游信号。但是，值得关注的是，抗EGFR抗体与TKI联合的毒副作用的叠加（多为皮肤病）可能会限制药物的长期使用。临床前模型试验显示，在多种细胞系中，这种联合会起协同作用^[75]^；但是，临床数据有限。一项Ⅰ期研究在难治性NSCLC患者中评估了西妥昔单抗联合吉非替尼的最佳剂量。研究结果证实这种联合方案的有效性适中（所有患者对该治疗均无反应，但31%的患者达疾病稳定）且耐受性较好；但是，据报道，11/13（84.6%）的患者出现治疗相关AE——痤疮样皮疹^[76]^。在研的以及今后的试验结果有望明确该具有疗效的联合方案在NSCLC中的可能应用。

## 其它靶向药物与抗表皮生长因子受体或抗血管内皮生长因子受体药物的联合

目前，作为NSCLC治疗性靶标的在研的其它生物学通路包括哺乳类动物雷帕霉素靶蛋白（mammalian target of rapamycin, mTOR），其为丝氨酸/苏氨酸激酶，是磷脂酰肌醇3激酶/Akt信号通路的下游调节因子，在细胞生长和增殖以及胰岛素样生长因子Ⅰ型受体（type Ⅰ insulinlike growth factor receptor, IGF-1R）通路的调节中起关键作用。目前，靶向作用于这些新型通路的制剂与抗EGFR或抗VEGF药物的合理联合作为NSCLC治疗选择正处于临床研发阶段。

在NSCLC中，mTOR通路中的信号被下调^[77]^，目前mTOR抑制剂雷帕霉素及其类似物[驮瑞塞尔（细胞周期抑制剂-779）、依维莫司（RAD001）和deforolimus（AP23573）]正处于癌症临床试验的评估中^[17]^。基于临床前数据^[8]^，EGFR与mTOR抑制剂的联合具有强有力的依据^[9, 10]^；最近一项有关厄洛替尼与依维莫司联合的Ⅰ期试验数据具有前景意义^[18]^。

IGF-1水平的升高与乳腺癌、前列腺癌、结肠癌和肺癌的危险性增加相关^[78]^，目前IGF-1R通路的抑制剂作为单一用药及与细胞毒性药物或其它靶向药物的联合尚处于临床研发中^[22]^。一项Ⅱ期试验显示，抗IGF-1R抗体联合化疗在晚期NSCLC中，尤其是在鳞癌细胞类型的患者中，疗效具有前景意义^[79]^。临床前数据也证实，IGF- 1R与EGFR信号通路间的交互作用可能参与NSCLC细胞系对TKI的耐受性的产生^[11]^。一项在研的Ⅲ期试验将评估厄洛替尼与IGF-1R通路抑制剂的联合治疗是否会引起NSCLC患者的有效性和生存期的改善^[25]^。mTOR抑制剂联合IGFR抑制剂已进行了临床前试验^[14]^，临床试验也已开始进行。

MET酪氨酸激酶为另一种细胞表面受体，可刺激细胞分散、侵袭、对细胞凋亡具有保护作用、血管形成。MET是靶向治疗干预的通用候选因子，单独或与其它靶向作用于多种通路（包括EGFR和VEGF通路）的药物联合以抑制MET的药物的临床研发目前正在进行中^[80]^。

血管破坏剂（vascular disrupting agent, VDA）是一类新型的癌症治疗药物，旨在通过引起异常内皮细胞凋亡和后继肿瘤细胞死亡以损害已经明确的为肿瘤提供营养的异常脉管系统^[81]^。多个VDA正处于临床研发中，大多数是与其它治疗方式联合，包括化疗、放疗以及其它靶向疗法（如，抗血管生成药物）。VDA可引起血管损伤并上调血管生成因子。这会导致循环中内皮细胞的增多，以及随后循环中内皮祖细胞（circulating endothelial progenitor cells, CEPs）的增多，这将导致肿瘤的耐受性。抗VEGF制剂与VDA的联合使用可能阻止CEP募集^[12]^，并有可能提高临床疗效。

## 总结

与标准化疗相比，分子靶向药物的合理联合有可能为晚期疾病患者提供额外的临床获益，最终也会降低毒副作用。EGFR和VEGF通路作为NSCLC治疗的靶标具有临床有效性，联合靶向作用于这些通路作为晚期NSCLC患者的治疗策略有强有力的生物学依据。通过多靶点药物或单一靶向药物的联合可靶向作用于这些通路；然而，一项有关晚期NSCLC的一线或二线治疗的随机Ⅲ期试验并未证实多靶点药物具有临床获益。在研的Ⅲ期试验有望为晚期NSCLC的多靶点治疗确定临床上更为有效且耐受性更好的方案。厄洛替尼联合贝伐珠单抗在晚期或转移性NSCLC的治疗中向前迈进了一步，为晚期NSCLC患者提供了一种非化疗且灵活的治疗方案。一些新型药物可阻断互补的信号通路，具有独特的作用机制，且毒副作用谱较小，这些新型药物的联合亦有可能会为晚期NSCLC患者的个体化治疗选择提供更广泛的治疗方案。

## Acknowledgment

Third-party medical writing support was provided by Genentech, Inc.; OSI Pharmaceuticals, Inc.; and F. Hoffmann-La Roche Ltd.

## Disclosures

Dr. Langer has received research funding from and has served as a paid consultant or been on the Advisory Board of ImClone Systems Incorporated and Pfizer Inc and is a member of the Speaker's Bureau for Bristol-Myers Squibb Company and ImClone Systems Incorporated. Dr. Soria has received consultancy fees from Abbot Laboratories, Amgen, AstraZeneca, Bristol-Myers Squibb Company, GlaxoSmithKline, Eli Lilly and Company, Pfizer Inc., Merck Serono International SA, MSD Pharmaceuticals, Roche Pharmaceuticals, sanofi-aventis U.S., and Wyeth Pharmaceuticals.
